# miR-17* Suppresses Tumorigenicity of Prostate Cancer by Inhibiting Mitochondrial Antioxidant Enzymes

**DOI:** 10.1371/journal.pone.0014356

**Published:** 2010-12-22

**Authors:** Yong Xu, Fang Fang, Jiayou Zhang, Sajni Josson, William H. St. Clair, Daret K. St. Clair

**Affiliations:** 1 Graduate Center for Toxicology, University of Kentucky, Lexington, Kentucky, United States of America; 2 Department of Radiation Medicine, University of Kentucky, Lexington, Kentucky, United States of America; 3 Department of Microbiology, Immunology and Molecular Genetics, University of Kentucky, Lexington, Kentucky, United States of America; 4 Cedars-Sinai Medical Center, University of California Los Angeles, Los Angeles, California, United States of America; University of Illinois at Chicago, United States of America

## Abstract

Aberrant micro RNA (miRNA) expression has been implicated in the pathogenesis of cancer. Recent studies have shown that the miR-17-92 cluster is overexpressed in many types of cancer. The oncogenic function of mature miRNAs encoded by the miR-17–92 cluster has been identified from the 5′ arm of six precursors. However, the function of the miRNAs produced from the 3′ arm of these precursors remains unknown. The present study demonstrates that miR-17* is able to suppress critical primary mitochondrial antioxidant enzymes, such as manganese superoxide dismutase (MnSOD), glutathione peroxidase-2 (GPX2) and thioredoxin reductase-2 (TrxR2). Transfection of miR-17* into prostate cancer PC-3 cells significantly reduces levels of the three antioxidant proteins and activity of the luciferase reporter under the control of miR-17* binding sequences located in the 3′-untranslated regions of the three target genes. Disulfiram (DSF), a dithiolcarbomate drug shown to have an anticancer effect, induces the level of mature miR-17* and cell death in PCa cells, which can be attenuated by transfection of antisense miR-17*. Increasing miR-17* level in PC-3 cells by a Tet-on based conditional expression system markedly suppresses its tumorigencity. These results suggest that miR-17* may suppress tumorigenicity of prostate cancer through inhibition of mitochondrial antioxidant enzymes.

## Introduction

Micro RNA (miRNA), ∼22 nucleotide RNA molecules that generally repress the translation of target messenger RNAs, is involved in various aspects of physiogenesis and pathogenesis [1]–[2]. The miR-17-92 cluster encodes six miRNAs, including miR-17, miR-18a, miR-19a, miR-20a, miR-19b-1, and miR-92, which are amplified in more types of cancer tissues than in corresponding normal tissues [3]–[5]. Proto-oncogene c-MYC up-regulates transcription of the miR-17–92 cluster and results in down-regulation of E2F1 by miR-17 [6], of PTEN by miR-19 [7], and of Rb1 by miR-20a [8], suggesting that miR-17–92 functions as an oncogenic factor. To date, the majority of miRNAs identified as having the ability to alter phenotype and development of cancer are generated from the 5′ arm of miRNA precursors. However, the production and function of the 3′ arm miRNA (miRNA*) remain elusive.

Deregulation of redox status is involved in a variety of pathogeneses including cancer. Reactive oxygen species (ROS) generated from oxygen metabolism is detoxified by multiple antioxidant pathways [9]. Mitochondrial antioxidant enzymes, including manganese superoxide dismutase (MnSOD), glutathione-dependent peroxidase (GPX) and thioredoxin- dependent peroxidase (TrxR2), comprise a primary defense system in mitochondria and are essential for detoxification against ROS [10]–[12]. A consequence of the high metabolism of rapidly growing cancer cells is the rapid generation of cellular ROS. Cancer cells therefore require a high antioxidant defense system to cope with the high levels of ROS production [13], [14]. Thus, selective inhibition of antioxidant systems is an option for cancer intervention.

Prostate cancer (PCa) is a common disease in North American males. Because PCa develops a castration-resistant phenotype, the levels of antioxidant proteins are increased and correlate to acquire capabilities, such as self-sufficient growth, reduced apoptosis, sustained angiogenesis, enhanced invasion and metastasis, as well as cancer cell resistance to treatment [15]–[17]. In this study, we use four complementary approaches to identify mediators for the tumor suppressing effect of miR-17*. The results demonstrate that suppression of mitochondrial antioxidant enzymes is a mechanism for the tumor suppressor function of miR-17*. This is the first report that reveals the link between oxidative stress and the tumor suppressor role of miRNA produced from the 3′ arm of the miR-17-92 cluster.

## Results

### miR-17* represses three mitochondrial antioxidant proteins

**Figure 1:**
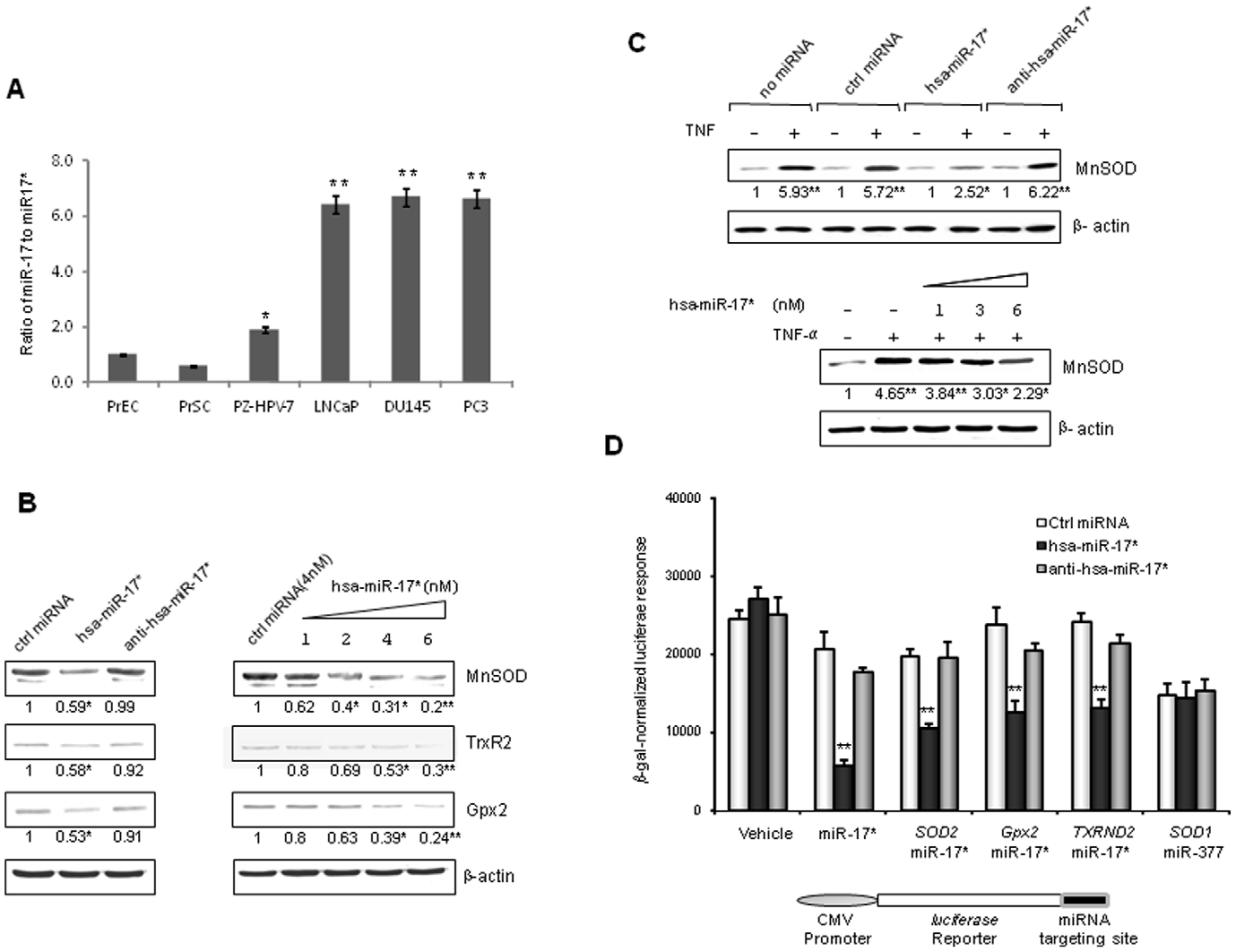
Identification of three mitochondrial antioxidant proteins as miR-17* targets. A, the levels of miR-17 and miR-17* expressed in PCa and control cell lines were measured by RT-PCR. The ratio of miR-17 to miR-17* in each cell line is presented. B and C, transfection of miR-17* in PC-3 cells to validate its function in repressing the expression of antioxidant proteins and diminishing TNF-mediated MnSOD induction. D, the repressive effect of miR-17* on the antioxidant proteins is estimated by quantitative luciferase reporter assay. RNU24 and β-actin were used as internal controls to normalize miRNA levels (A), protein levels (B and C). Images were normalized with the internal controls and then normalized by PrEC (A), by control miRNA (B), and by no TNF treatment (C). β-gal activity was used to normalize luciferase reporter activities (D). Three samples (n = 3) were used in the experiments and fold changes in Western blots are indicated. * (p<0.05) and ** (p<0.01) indicate significances as compared to the controls: PrEC (A), control miRNA (B) and (D), and untreated samples (C).

A number of studies have reported that miR-17 is highly expressed in malignant tumors including PCa, but the level of its partner, miR-17*, is normally low in cancers [18]. This evidence predicts that the levels of miR-17 and miR-17* are differentially regulated and that the ratio of the two miRNAs may be important for regulation of different sets of target genes during tumorigenesis. The expressions of both miRNAs in different prostate cells, including normal epithelial, stromal, viral transformed, androgen-responsive and androgen-independent PCa cells, were quantified by real-time RT-PCR. The level of miR-17 and the ratio of miR-17 to miR-17* in PCa cells were higher than levels in non-cancerous cells (Fig. 1A). By searching miRbase, we found that MnSOD, Gpx2 and TrxR2, three important mitochondrial antioxidant proteins that are essential for detoxification of O_2_^.−^ and H_2_O_2_, are potential targets of miR-17*. To verify that miR-17* is able to repress antioxidant proteins, mature miR-17* was transfected into PC-3 cells, which have a low level of endogenous miR-17*. The expression of the three antioxidant proteins was reduced by the transfected miR-17* in a dose-dependent manner. Whereas transfection of control miRNA and antisense miR-17* had no effect on the targets (Fig. 1B), oxidative stress stimuli such as cytokines are able to induce the expression of the *SOD2* gene through activation of the NF-κB signaling pathway [19]. Transfection of miR-17* significantly repressed TNFα-induced *SOD2* expression in a dose dependent manner (Fig. 1C). To confirm that the reduction of antioxidant proteins by miR-17* is mediated through translational repression, the 3′- untranslated regions, including the putative miR-17* targeting sites of the genes coding for the three antioxidant enzymes, were cloned downstream of the reporter gene. A vehicle and a miR-377 targeting site located in the 3′untranslated region of the *SOD1* gene were included as vehicle control and nonself negative control. As shown in Fig. 1D, the cloned miR-17* targeting sequences are necessary for the repression of reporter responses by transfected miR-17*.

### DSF induces miR-17* expression

**Figure 2:**
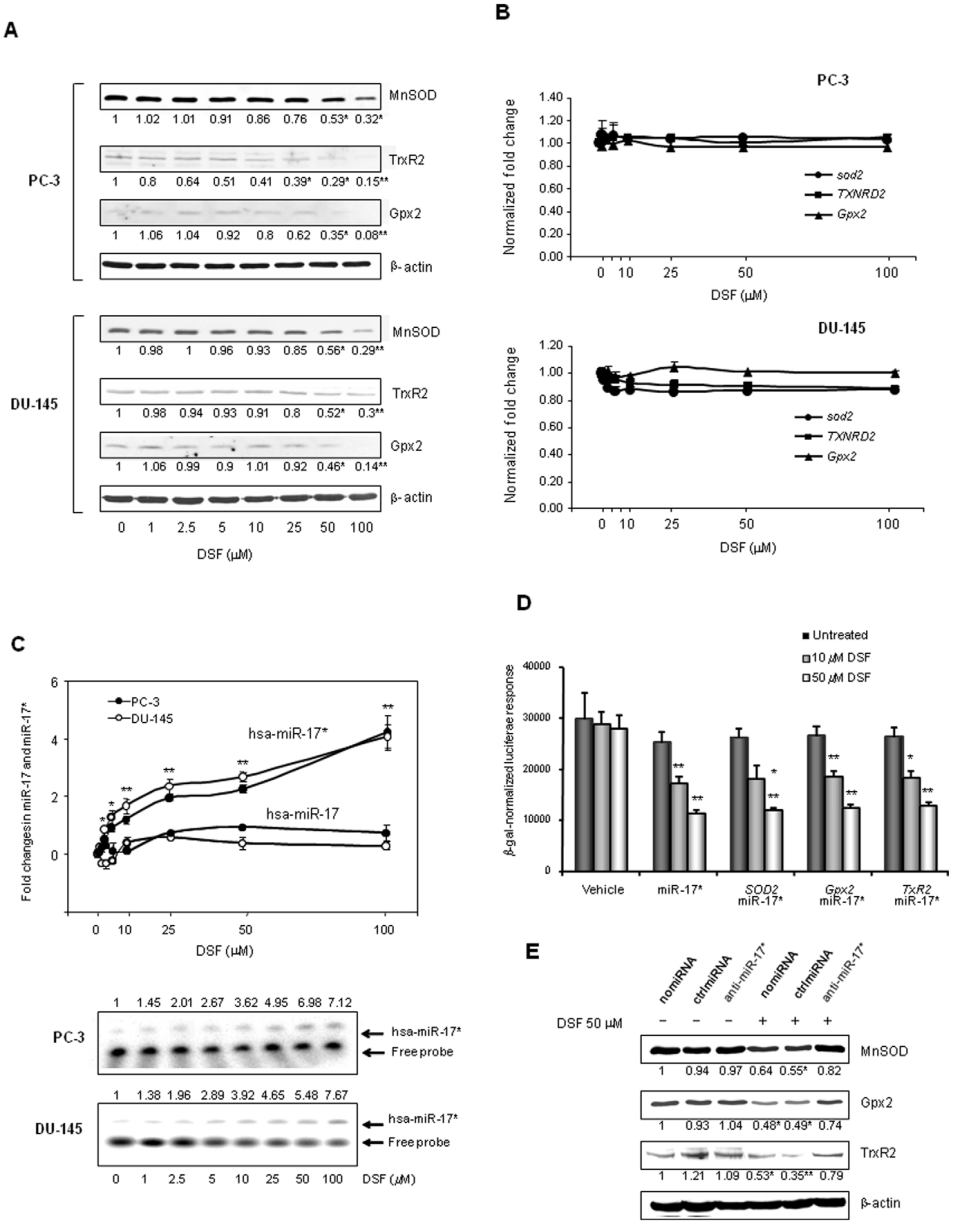
Induction of miR-17* in PCa cells by DSF. A, PCa cells were treated with DSF at indicated concentrations. The levels of the three antioxidant proteins were measured by Western blots. B, mRNA levels of the three antioxidant genes were quantified by RT-PCR. C, the levels of miR-17 and miR-17* in the DSF-treated cells were quantified by RT-PCR. The miR-17* levels were confirmed by Northern blots. D, the effect of DSF-induced miR-17* on the reporter responses was determined. E, after transfected anti-miR-17*, PC-3 cells were treated with DSF. The effect of anti-miR-17* on restoring antioxidant proteins was quantified by Western blots. β-actin was used to normalize the levels of proteins (A), (E), and the levels of mRNA (B). The fold changes are indicated. RNU24 was used to normalize the levels of miR-17 and miR-17* (C). β-gal activity was used to normalize luciferase reporter activities (D). Three samples (n = 3) were used in the experiments (with the exception of Northern blot). * (p<0.05) and ** (p<0.01) indicate significances as compared to no DSF treatment (A), (C), (D), and compared to no DSF and no miRNA transfected samples (E).

DSF is a dithiolcarbomate drug that has been shown to suppress cancerous phenotypes by inducing the apoptotic pathway [20]. We found that DSF inhibits the three antioxidant proteins in PCa cells. After PC-3 and DU-145 cells were treated with DSF for 24 h, the reduction of the levels of the antioxidant proteins corresponded significantly with the concentrations of DSF (Fig. 2A). However, the mRNA levels of the antioxidant genes were not changed in DSF-treated cells (Fig. 2B). Interestingly, RT-PCR and Northern blots show that DSF induces miR-17* but has no effect on expression levels of miR-17 (Fig. 2C). The induction of miR-17* by DSF is further confirmed by reporter responses that are regulated by miR-17* targeting sequences (Fig. 2D). Furthermore, to verify that the negative effect of DSF on the expression of antioxidant proteins is mediated by the induction of miR-17*, the PC-3 cells were transfected with antisense hsa-miR-17* followed by DSF treatment. The results indicate that the antisense hsa-miR-17* is able to reduce the DSF effect (Fig. 2E). These results suggest that the reduction of antioxidant proteins by DSF occurs, at least in part, through miR-17*-mediated translational repression.

### miR-17* induces cell death in PCa cells

**Figure 3:**
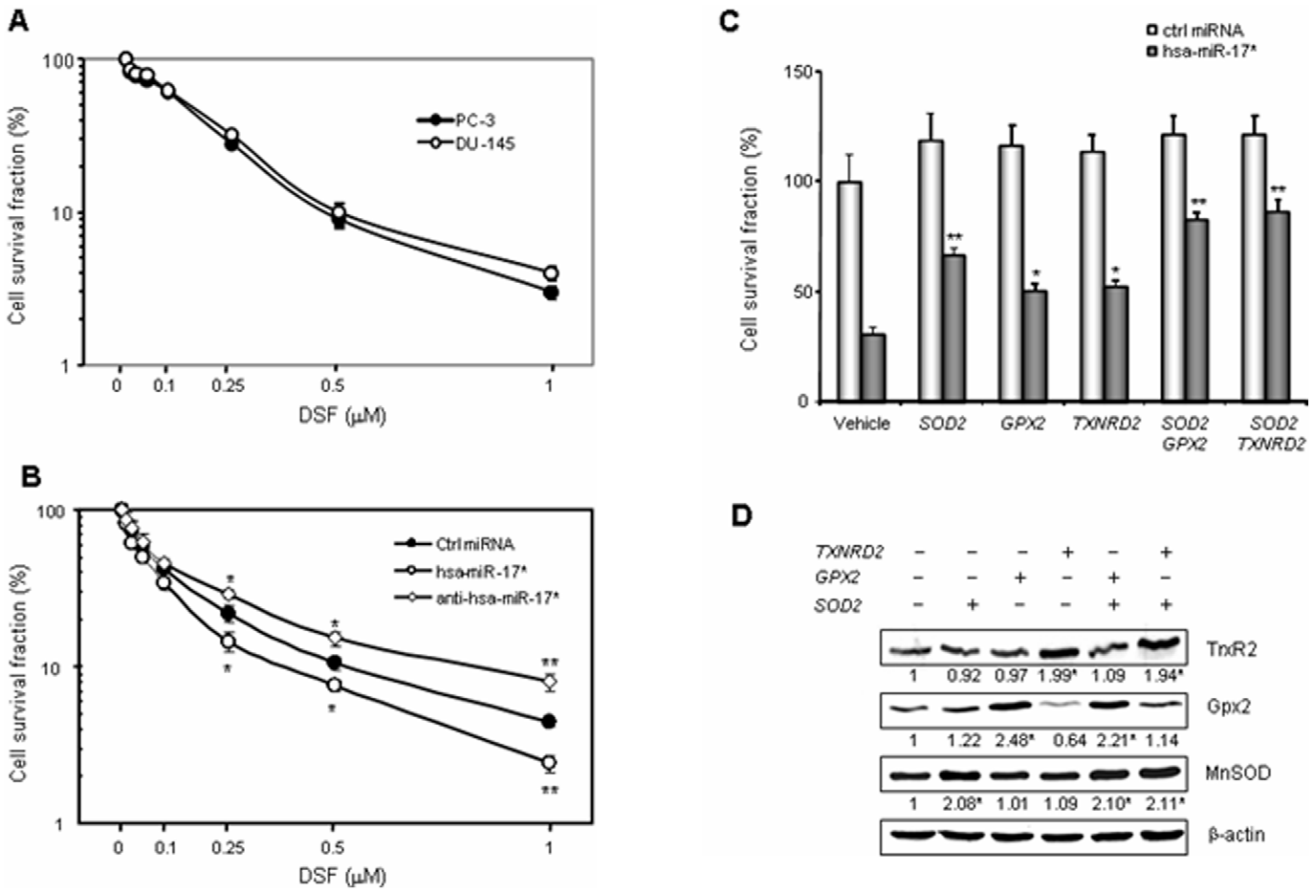
Cytotoxicity of miR-17* in PCa cells. A, the PCa cells were treated with DSF at the indicated concentrations for colony survival analysis. The formed colonies were counted and plotted in a log scale. B, the PC-3 cells were transfected with miR-17* and control miRNAs prior to the DSF treatment. The effects of miR-17* and antisense miR-17* on colony survival were determined. C and D, miR-17* was co-transfected with constructs for expression of the three antioxidant proteins. The overexpressed antioxidant proteins were confirmed by Western blots with β-actin normalization and fold changes are indicated (D). Protective effects of the transfected antioxidant enzymes on the cells against miR-17* toxicity were determined by a trypan blue exclusion assay (C). Three samples (n = 3) were used in the experiments. * (p<0.05) and ** (p<0.01) indicate significances as compared to control miRNA samples (B) and compared to vehicle control samples (C), (D).

To determine DSF toxicity to PCa cells, PC-3 and DU-145 cells were treated with DSF and cultured until colonies formed. Because the cell density used for colony formation analysis was 100-fold less than the density shown in Fig. 2, the concentration range of DSF was reduced 100-fold in colony formation experiments. The results of survival fraction indicate that PCa cells are extremely sensitive to DSF. More than 95% of the cells were killed by treatment with 1 µM DSF (Fig. 3A). To verify whether miR-17* contributes to DSF-mediated cell death, PC-3 cells were transfected with miR-17* and anti-miR-17* prior to DSF treatment. Colony survival analysis shows that miR-17* enhances the toxicity of DSF, whereas anti-miR-17* is able to rescue cells from the DSF effect (Fig. 3B). To further verify that reduction of antioxidant proteins is a major cause for the toxicity of miR-17*, the PC-3 cells were co-transfected with miR-17* and cDNAs ectopically expressing the three antioxidant proteins that are not susceptible to miR-17* regulation. Cytotoxicity analysis by trypan blue exclusion assay shows that expression of the three antioxidant genes rescues cell survival from the toxicity of miR-17* (Fig. 3C). The corresponding levels of the antioxidant proteins in the transfected PC-3 cells were verified by Western blots (Fig. 3D).

### miR-17* suppresses the tumorigenicity of PCa

**Figure 4:**
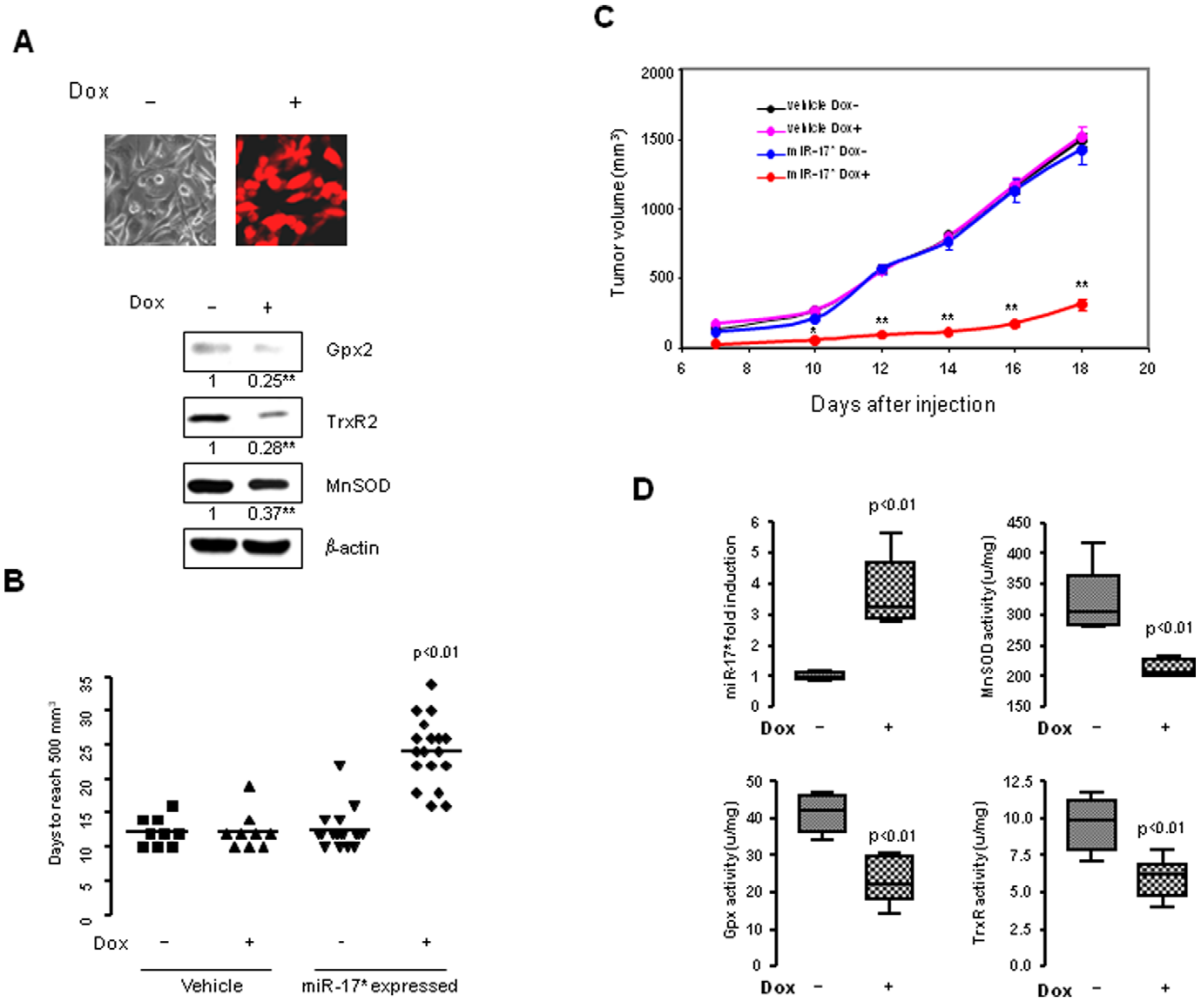
Suppression of tumorigenicity of PC-3 by expression of miR-17*. A, has-miR-17* was cloned in a Tet-on lentiviral vector and stably transected into PC-3 cells. The clone was tested by RFP screening under Dox- inductive conditions and then confirmed by measuring the expression of the three target genes using Western blots with β-actin normalization. B and C, the generated clone was injected into male nude mice to determine its tumorigenicity. The vehicle control was included. The number of days needed for tumor size to reach 500 mm^3^ is shown in (B) and calculated tumor growth rates in (C). D, total RNA and proteins were isolated from the tumor tissues and the level of miR-17* and corresponding activities of the three antioxidant proteins were quantified. Three samples (n = 3) were used in testing the generated miR-17* inducible clone (A). Nine vehicle control animals (n = 9) with or without DOX treatment and eighteen miR-17* expressed animals (n = 18) with or without DOX treatment were used to test the effect of miR-17* on tumor growth (B), (C), (D). * (p<0.05) and ** (p<0.01) indicate significances as compared to without DOX control (A) and (C).

To determine the effect of miR-17* on tumor growth, the sequence of mature miR-17* was cloned into a Tet-on based lentiviral expression vector. The lentivirus expressing miR-17* was transducted into PC-3 cells, and a stable cell line with Tet-on based RFP expression was identified. The effect of miR-17* on the three antioxidant proteins under Tet-on inducible conditions was confirmed by Western blots (Fig. 4A). A mouse xenograft tumor model was used to evaluate the effect of miR-17* on tumor growth. When the clone was subcutaneously injected into nude male mice, expression of miR-17* *in vivo* was induced by administering Dox containing water. A vehicle control was included to control the toxic effect of Dox. As shown in Fig. 4B, the average time for tumors to reach 500 mm^3^ in the vehicle control and miR-17* without Dox control groups is 12 to 13 days (vehicle control without Dox, 12.2±2.1; vehicle control with Dox, 12.3±2.8; and miR-17* without Dox, 12.6±2.8). Notably, the number of days for the miR-17* with Dox group to reach 500 mm^3^ tumor size is 24±4.9, which is twice as long as the control groups. To continuously measure tumor growth, mice in the control groups were kept for 18 days after injection when tumor size reached the maximum allowable size of 2000 mm^3^. The tumor growth rates shown in Fig. 4C indicate that the tumor growth in the miR-17* with Dox group was significantly delayed as compared to the tumor growth in the control groups. To verify whether the expression of miR-17* results in reduced antioxidant proteins in the miR-17* expressed tumor tissues, the levels of miR-17* and activities of the antioxidant enzymes in the tumor tissues were quantified. Corresponding to the increased levels of miR-17* in the Dox-treated group, the activities of the three antioxidant enzymes were significantly reduced as compared to the untreated group (Fig. 4D). Taken together, these results demonstrate that the expression of miR-17* in PC-3 cell reduces the tumorigenicity, at least in part, by inhibiting mitochondrial antioxidant function. This result suggests that in contrast to the oncogenic effect of miR-17, miR-17* plays a tumor suppressive role in PCa cells.

## Discussion

miRNA generally functions as a posttranscriptional repressor, which is thought to be an important mechanism of gene regulation. miRNA biogenesis includes canonical primary miRNA transcription, Drosha/Dicer-mediated cleavages, and strand preferential selection through Argonaute (AGO) proteins [21]. When one strand is selected for repression of targets, its partner strand is presumed to be degraded [22]. However, recent studies have detected both miR-17 and miR-17* in many types of human tissues [23]. In all cell lines tested, our results demonstrate that miR-17* is present at a lower level than miR-17. However, the levels of both miRNAs are higher in PCa cells than in the control cells (data not shown). Since the level of miR-17 is higher than miR-17*, the ratio of miR-17 to miR-17* in PCa cells is increased, suggesting that miR-17 is a preferentially selected strand, although both miR-17 and miR-17* precursors are transcribed. Recent studies have demonstrated that AGO1 mediates miRNA production in Drosophila, while AGO2 is associated with miRNA* production [24]. However, the precise mechanism by which AGO regulates miRNA biogenesis needs to be determined to uncover preferential accumulation of miRNA strands under different conditions.

Our data demonstrate that miR-17* suppresses tumorigenicity of PCa cells, suggesting that the function of miR-17* is opposite to the oncogenic function of miR-17. Expression of the miR-17-92 cluster is tightly regulated in response to intercellular and extracellular environments. Transcription of this cluster is up-regulated by c-Myc under oxidative conditions [25] and down-regulated by p53 under hypoxia conditions [26]. Interestingly, DSF, a dithiolcarbomate, induces only the level of miR-17* and not miR-17. This selective induction is consistent with the tumor suppressive effect of miR-17* and is in agreement with a previous finding that DSF induces apoptosis in cancer cells [20]. Taken together, these results suggest that DSF may be effective as an anticancer agent, in part by induction of miR-17*.

miRNA-based gene repression is considered to be a crucial regulator controlling cell fate. However, it is a complicated regulation system because one gene can be regulated by multiple miRNAs and one miRNA has many different targets. In general, the effect of miRNA on gene regulation is dependent on specific tissue types, development statuses, or stimuli. Thus, identification of miRNA targets is critical to define the real functions of miRNA under physiological or pathological conditions. Our study demonstrates that miR-17* is a negative regulator for three important antioxidant enzymes located in mitochondria. These antioxidant enzymes are major components of the primary antioxidant system and they work in concert to safely remove ROS generated in mitochondria. Inhibition of these proteins should lead, therefore, to an accumulation of ROS, resulting in cytotoxicity. Our findings suggest a novel therapeutic approach to enhance cell death by miR-17* targeting. In addition, a recent study demonstrated that miR-17 is able to silence HIF-1α expression, a transcription factor for maintenance of redox homeostasis and cell survival under hypoxic conditions [27]. Together, these findings predict that the ratio of miR-17 to miR-17* may have an important role in the regulation of cellular redox status.

Although the Warburg effect, a high rate of aerobic glycolysis in tumors, has been observed in various types of cancer, cancers have functional mitochondria and mitochondrial respiration is necessary for cancer cell proliferation [28]. Furthermore, cancer cells have high levels of ROS and also express high levels of antioxidant proteins to detoxify the elevated rates of ROS generation [29]. For instance, MnSOD is expressed at a high level in aggressive PCa cells, which is essential for protection of PCa cells against radiation-induced ROS [30]. Thus, triggering pro-apoptotic signaling pathways by targeting mitochondria antioxidant enzymes could also be considered an anti-cancer therapeutic strategy. Our results, which demonstrate that miR-17* inhibition of mitochondrial antioxidant proteins suppresses tumorigenicity of PCa cells in vivo, provide experimental evidence for the proof-of-concept that miRNA* can function as a tumor suppressor by inhibition of mitochondrial defense capacities.

## Materials and Methods

### Cell culture and cell toxicity analysis

PrEC, human prostate epithelial primary cells (Cambrex Corp.), and PrSC, human prostate stromal cells (Clonetics), were grown in PrEBM medium (Lonza). PZ-HPV-7, HPV-18 transformed human prostate epithelial cells (American Type Culture Collection, ATCC), was grown in Keratinocyte-SFM medium (Invitrogen). Human epithelial carcinoma cells LNCaP, DU-145 and adenocarcinoma PC-3 cells (ATCC) were grown in RPMI medium (Invitrogen) containing 10% FCS (Hyclone). Colony formation assay was used to quantify toxicity of miR-17* to PCa cells plated in 6-well plates at low densities. To induce miR-17* expression, the PCa cells were treated with DSF at a concentration range of 0 to 1 µM for 24 h. The colonies were washed with 1x PBS and stained with a crystal violet dye. The surviving fraction was calculated as the ratio of the number of colonies formed to the number of cells efficiently plated. Trypan blue exclusion assay was used to determine the protective effects of increased antioxidant enzymes on the toxicity of miR-17*. The cells were co-transfected with miR-17* and expression constructs of the three antioxidant genes. After culture for 48 h, the transfected cells were stained with a 0.4% trypan blue dye and counted using a Vi-Cell cell viability analyzer (Beckman Coulter).

### Micro RNA expression reporter assay

To test whether miR-17* regulates expression of the target genes, the 3′-untranslated regions of the target genes containing the putative miR-17* binding sites were cloned between *Sac* I and *Hind* III sites of the pMIR-reporter vector (Ambion). The generated miRNA expression reporter constructs were co-transfected with β-gal internal control vector (Ambion) into PC-3 cells using lipofectamine (Invitrogen). After culture for 36 h, the cells were harvested; luciferase activity was measured using a luciferase assay kit (Promega); and β-gal activity was measured using chlorophenol red-a-D-glactopyranoside monosodium substrate (Roche Molecular Biochemicals). Relative luciferase responses were estimated by β-gal-normalized luciferase activity.

### Expression of miR-17*

To increase miR-17* level in PCa cells, miR-17* molecules and controls (Ambion) were transfected into the cells using oligofectamine (Invitrogen). To induce miR-17* expression in PCa cells, the cells were treated with disulfiram (Sigma) at a concentration of 0 to 100 µM for 24 h. To stably express miR-17* in PCa cells, a mature miR-17* sequence spanned by Drosha and Dicer cleavage sites was cloned into a Tet-on inducible lentiviral vector, TRIPZ (Open Biosystems), using Xho I and EcoR I sites. Sequence of insert containing the miR-17* (shown by an underline) is CTCGAGTGCTGTTGACAGTGAGCGAACTGCAGTGAAGGCACTTGTAGTAGTGAAGCCACAGATGTACTACAAGTGCCTTCACTGCAGTCTGCCTACTGCCTCGGAGAATTC. The cloned miR-17* was packaged using a translentiviral packaging system (Open Biosystems). The miR-17* lentivirus was concentrated and titered prior to transduction into the cells under 2 µg/ml puromycin selective conditions. The miR-17* clone was further selected by Tet-on inducible expression of a red fluorescence protein (RFP) tag using media containing 1 µg/ml doxycycline (Dox). The stable clone was verified by screening the expression of the targets using Western blots.

### Expression of antioxidant enzymes

To rescue cell survival from the toxicity of miR-17*, cDNA constructs for expression of the three antioxidant proteins were transfected into PC-3 cells prior DSF treatment. The ectopically expressed antioxidant proteins are not affected by has-miR-17*, because the cDNA constructs do not have the 3′-untranslational regions where the binding sites are identified for has-miR-17* binding.

### Animals

Four-weeks-old male NCRNU (nu/nu athymic nude) mice were purchased from Taconic (Hudson, NY). 10^6^ cells mixed with Matrigel (BD Biosciences) were injected into the right flank of the mice. The injected mice were separated into two groups: two days before injection, one group of mice started to drink water containing 2 mg/L doxycycline and the control group continued to drink regular water. Tumor volumes were calculated using a standard formula (A×B^2^×.52; A and B represent the diagonal tumor lengths).

### Western blots

Proteins were extracted from cultured cells and tumor tissues as described previously (11) and 100 µg of extracted proteins were electrophoresed on an 8% (w/v) SDS-PAGE gel, transferred onto a nitrocellulose membrane, and subsequently incubated with primary antibodies against MnSOD (Upstate Biotech.), Gpx2 (Abcam), TrxR2 and β-actin (Santa Cruz Biotech). Western blots were visualized using an enhanced chemiluminescence detection system (ECL, Amersham Pharmacia Biotech.).

### Real-time PCR (RT-PCR)

To enrich miRNA in RNA preparation, total RNA was isolated from the cultured cells and tumor tissues using a mirVana miRNA Isolation Kit (Ambion). For quantification of miR-17 and miR-17*, the RNA was analyzed using a TaqMan MicroRNA Reverse Transcription Kit with internal controls RNU6B, RNU24 and RNU48 (Applied Biosystems). To quantify mRNA levels of the miR-17* target genes, the RNA was analyzed using TaqMan Reverse Transcription Reagents (Applied Biosystems) and RT-PCR with the Universal ProbeLibrary Set (Roche Applied Science). RT-PCR was performed in a TaqMan Universal PCR Master Mix using a LightCycler 480 Real-Time PCR System (Roche Applied Science).

### Northern Blots

The level of miR-17* was quantified using a miRtect-IT miRNA Labeling and Detection kit (USB Corp.) in accordance with the manufacturer's protocol.

### Enzyme activity assay

MnSOD activities were measured by the nitroblue tetrazolium (NBT)-bathocuproin sulfonate (BCS) reduction inhibition method. Sodium cyanide (2 mM) was used to inhibit Cu/ZnSOD activity [31]. GPx activity was measured by using a reaction mixture consisting of 0.2 mM H_2_O_2_, 1.0 mM GSH, 0.14 U of glutathione reductase (GR), 1.5 mM NADPH, 1.0 mM sodium azide, and 0.1 M phosphate buffer (pH 7.4) and 1 mg/ml of supernatant protein [32]. TrxR activity was measured using Thioredoxin Reductase Activity Assay Kit (Redoxica) in accordance with the manufacturer's protocol.

### Statistical data analyses

Multiple independent experiments were performed for each set of data. Images in Northern blots and Western blots were quantified using Carestream Molecular Imaging software (Carestream Health Inc.). Statistical significance was analyzed using one-way ANOVA and Tukey's Multiple Comparison Test, followed by data analysis with Graphpad Prism.
